# Non-targeted analysis with high-resolution mass spectrometry for investigation of riverbank filtration processes

**DOI:** 10.1007/s11356-022-20301-2

**Published:** 2022-04-26

**Authors:** Kaan Georg Kutlucinar, Sebastian Handl, Roza Allabashi, Tim Causon, Christina Troyer, Ernest Mayr, Reinhard Perfler, Stephan Hann

**Affiliations:** 1 Department of Chemistry, Institute of Analytical Chemistry, University of Natural Resources and Life Sciences, Vienna, Muthgasse 18, 1190 Vienna, Austria; 2 Department of Water, Atmosphere and Environment, Institute of Sanitary Engineering and Water Pollution Control, University of Natural Resources and Life Sciences, Vienna, Muthgasse 18, 1190 Vienna, Austria

**Keywords:** Riverbank filtration, High-resolution mass spectrometry, Non-targeted analysis, Surface water/groundwater interactions, Solid-phase extraction

## Abstract

A fully non-targeted analytical workflow for the investigation of a riverbank filtration site located at the river Danube has been developed and applied. Variations of compound intensities at different sampling locations of the riverbank filtration site and, for a single production well, over a monitoring period of one year have been investigated using liquid chromatography combined with time-of-flight-mass spectrometry followed by evaluation via non-targeted data analysis. Internal standardization and appropriate quality control strategies have been implemented into the workflow for reduction of possible methodological biases influencing data interpretation. Emphasis was placed on the assessment of different blank elimination steps and the final blank elimination strategy is reported. The spatial study of the selected riverbank filtration site revealed a homogenous composition of the filtered water sampled at 11 different locations across the 32,000 m^2^ site, except for one sampling location in a zone of the aquifer, which was only weakly connected to the well field in terms of hydrogeological conditions. The examination of time-dependent changes of the composition of surface and groundwater obtained at the riverbank filtration system revealed that the non-targeted workflow is fit-for-purpose regarding the assessment the stability of filtration efficiency and compound residence time in the riverbank filtration compartment. In total, 677 compounds were selected for the investigation of the time-dependent variations of the filtration process. Analysis of the signal intensities of these compounds revealed that the riverbank filtration is significantly reducing the intensity and number of compounds present in surface water over a wide polarity range. In addition, the method enabled the determination of compound residence times in the riverbank filtration system ranging from 5 to 7 days.

**Supplementary Information:**

The online version contains supplementary material available at 10.1007/s11356-022-20301-2.

## Introduction

The increasing production and use of industrial organic chemicals such as pesticides, personal care products, artificial food additives, pharmaceuticals, corrosion inhibitors, and flame retardants result in continuous contamination of the environment (Postigo and Barceló 2015; Hollender et al. 2017). The release of emerging organic contaminants, defined as unregulated organic contaminants, into the aquatic environment via discharge of municipal, hospital, and industrial wastewater effluents, agricultural run-off, combined sewage-storm-water overflows, and waste disposal sites adds a new challenge in ensuring the supply of safe drinking water for the increasing population (Kemper 2008; Verlicchi et al. 2010; Houtman 2010; Klamerth et al. 2013; Pal et al. 2014; Petrie et al. 2015; Schaider et al. 2017; Thomaidis et al. n.d).

Safe drinking water can be produced using different water sources including surface water, groundwater, bank filtrate, reservoir water, and spring water (Kuehn and Mueller 2000). Depending on the nature of the water source, a range of different treatments steps need to be applied in order to meet safe drinking water quality requirements. In this context, riverbank filtration represents a natural process allowing a lower number of required treatment steps to produce safe drinking water. Riverbank filtration has been used in several European countries for more than 100 years for drinking water production and can replace conventional water treatment steps such as coagulation, flocculation, and sedimentation, thereby reducing water processing costs (Kuehn and Mueller 2000; Tufenkji et al. 2002; Grünheid et al. 2005). In order to achieve efficient filtration in this setting, water is withdrawn from production wells that are placed adjacent to an adequate surface water body (e.g., rivers, lakes, or basin). The withdrawal of water induces a hydraulic gradient that forces the surface water to flow through the bed and banks of the surface water body to the production well (Ray 2011). The water flow through the riverbed sediments and aquifer materials improves the water quality by filtering most suspended and dissolved contaminants, including disinfection by-product precursors, pathogenic bacteria, and viruses. In addition to physicochemical and biological processes such as precipitation, sorption, redox reactions, and metabolic processes of aerobic and anaerobic microorganism, also, the dilution caused by mixing with groundwater improves the water quality by reducing the concentration of pollutants (Ray et al. 2002; Weiss et al. 2005; Ray 2008; Hoppe-Jones et al. 2010; Rossetto et al. 2020). Most of the natural filtration of fine sediments, particulate organic matter, and pathogens occurs within the first few meters from the river to the well (Jaramillo 2012).

Investigations of riverbank filtration systems have shown promising results concerning the reduction of total organic carbon (TOC), dissolved organic carbon (DOC), natural organic matter, and emerging organic contaminants from surface water (Ludwig et al. 1997; Hoppe-Jones et al. 2010; Benotti et al. 2012; Ahmed and Marhaba 2017). With the development of analytical methods providing quantitation limits lower than 1 µg/L, the fate of emerging organic contaminants in riverbank filtration could be investigated in more detail. The removal efficiency of emerging organic contaminants such as carbamazepine, clofibric acid, sulfamethoxazole, and amidotrizoic acid was varying depending strongly to the underlying redox conditions. However, the influence is not uniform. While some compounds such as carbamazepine and amidotrizoic acid were more degradable under anaerobic conditions, other compounds such as fenofibric acid and naphthalene-1,6-disulfonate were more strongly degraded under aerobic conditions. Recent research demonstrated that riverbank filtration does not represent a generic elimination system for all emerging organic contaminants, but it can lower significantly the concentration of such compounds in surface water (Schmidt et al. 2007; Massmann et al. 2008; Heberer et al. 2008).

The performance of riverbank filtration can be affected by the pollution load of surface water, flow velocity and bedload characteristics, and stability of the river channel (Hunt et al. 2003). Hence, depending on the efficiency and spatiotemporal stability of the riverbank filtration process, the filtered water may still require additional treatment before meeting the regulations for safe drinking water (Kuehn and Mueller 2000; Ray et al. 2002). Current literature indicates that theoretical prediction of riverbank filtration efficiency and resulting water quality is, despite the tremendous progress in modeling, still very challenging. Consequently, precise and comprehensive analytical data on the biological and chemical state of the filtered water is needed for the assessment of the filtration process (Jaramillo 2012).

LC combined with HRMS is currently recognized as the most suitable compromise for the detection of a wide range of polar and moderately polar compounds present in water samples, as samples can be directly injected or pre-concentrated without any prior chemical treatment (Krauss et al. 2010; Richardson and Ternes 2011; Reemtsma et al. 2016; Borrull et al. 2019). After the data acquisition by HRMS systems (time-of-flight (TOF)MS, Orbital ion trap MS, Fourier transform (FT)MS), which is usually performed over a wide mass range in the so-called MS1 (or full-scan) mode, data evaluation can be conducted applying three different strategies: (i) targeted data analysis: software-based extraction of the exact monoisotopic mass and isotopologue pattern of selected compounds in a predefined retention time interval derived from measurement of a reference standard (Schymanski et al. 2015). This type of analysis is providing the highest confidence level with regard to compound identification, i.e., level 1 (Schymanski et al. 2014). Furthermore, absolute quantification of the preselected compounds is possible if calibration with authentic standards has been performed. Evidently, this strategy is limited as full coverage of all compounds of interest would require the purchase or synthesis of a vast number of reference substances (Hollender et al. 2014); (ii) suspect screening (also called “non-target screening”): software-based extraction of the exact monoisotopic mass and isotopologue pattern of suspect compounds from the acquired datasets, without using comparative measurements of authentic standards. Together with HRMS fragment spectra obtained in the same run, or from additional analytical runs, this strategy enables tentative identification of compounds, i.e., level 2 (Schymanski et al. 2014), and is also used for retrospective analysis following the identification of new suspects of interest (López et al. 2014; Hollender et al. 2014, 2017). (iii) Non-targeted data analysis: MS1 data is processed and evaluated in a workflow where peak picking and expected ion associations (e.g., isotopologues, adducts, dimers) are aligned to find potential compounds of interest. Compared to suspect screening approaches, fully non-targeted data processing does not extract known monoisotopic masses but applies a peak picking algorithm, which yields a list of compounds designated with a retention time, accurate monoisotopic mass, associated ions, respective spectral abundances, and supplementary fragmentation information if available. This strategy is most often used to perform differential analysis of samples or sample groups via relative quantification where compound abundance is considered as a proxy for the amount (Nürenberg et al. 2015). Generally, targeted data analysis and suspect screening are fit-for purpose for, e.g., pollutant monitoring, whereas the added value of non-targeted data analysis lies in its potential of compound discovery, including unknown transformation products. Non-targeted analysis has been developed and applied for evaluation of water treatment processes, e.g., ultraviolet radiation (Merel et al. 2015), advanced oxidation reactor (Parry and Young 2016), ozonation (Schollée et al. 2021), and riverbank filtration (Hollender et al. 2018; Oberleitner et al. 2020). These investigations have shown that riverbank filtration is a natural drinking water treatment procedure with a potential to reduce and remove soluble organic micro contaminants.

Following MS1-based HRMS acquisition, an identity confirmation workflow can be used with confidence increasing according to the matching quality of accurate mass, isotopologue pattern, adducts, and fragmentation information. However, in order to achieve the highest degree of confidence in this workflow (level 1), measurement of a reference standard with MS1, MS2, and retention time matching is needed (Schymanski et al. 2014). Despite major efforts toward improving the range of identified pollutants for use in spectral libraries, the identity confirmation of true unknowns is a laborious task and remains impossible for a large amount of unknown compounds due to several factors including the range of possible isomers, non-unique fragment spectra, in-source fragmentation, and a lack of standardization for chromatographic and MS2 fragmentation within existing libraries (Zedda and Zwiener 2012; Hollender et al. 2017). Thus, before commencing with an identity confirmation workflow, the usually high number of detected compounds should be reduced to a selection of compounds of interest according to the original research hypothesis. This selection can be achieved by prioritization approaches considering, e.g., signal intensity (Hollender et al. 2017), fold changes, frequency of occurrence, and through other statistical analysis methods.

This work represents a proof-of-concept study testing LC-TOFMS-based non-targeted analysis for the investigation of a riverbank filtration system located at the river Danube in Vienna, Austria. NTA was employed for comprehensive characterization of a riverbank filtration site both in terms of spatial and temporal variations of the filtration process. First, the spatial variability of 11 wells in a 32,000-m^2^ riverbank filtration site was characterized. Second, the time-dependent variability of surface water and the corresponding riverbank filtrate obtained from a single production well was monitored at 25 time points over the period of one year. Combined results allowed to characterize riverbank filtration in terms of the influence of groundwater sampling location to the reduction/increase of the content of organic compounds and estimation of the residence time of selected compounds in the riverbank filtration compartment.

## Material and methods

### Chemicals

LC–MS grade water, methanol (MeOH), and 2-propanol (IPA) were purchased from Honeywell (Chromasolv™ HPLC solvents series, Bucharest, Romania). Formic acid (FA; 98–100%, Suprapur®) was purchased from Merck Millipore (Darmstadt, Germany). Norleucine (98% purity), naringenin (98% purity), nicotinamide (≤ 99.5% purity), and ααα-trifluoro-*m*-toluic acid (99% purity) were purchased from Sigma-Aldrich (Vienna, Austria). Ultrapure water for pre-cleaning of apparatuses was prepared with arium® pro UV ultrapure water system (Sartorius, Goettingen, Germany) equipped with arium® analytical kit (Sartorius) and Sartopore® 2 capsule 0.2 µm (Sartorius).

### Preparation of internal standards and quality control samples

Two different internal standards were prepared. Standard A contained nicotinamide and ααα-trifluoro-*m*-toluic acid at a concentration of 8.5 µmol L^−1^ and was prepared by dissolving the solid substances in MeOH and dilution with MeOH. Standard B contained norleucine and naringenin at a concentration of 4 µmol L^−1^ and was prepared by dissolving the solid substances in MeOH and dilution with MeOH.

For each batch, pooled quality control (QC) samples were prepared by blending 3 or 4 water samples, which were randomly selected from the corresponding sample batch. For each study, technical replicates of corresponding QC sample were measured (replicates from the same HPLC vial).

### Pre-cleaning of apparatuses

All glassware used for sample preparation except elution tubes was washed in a laboratory dishwasher (Miele, Wals, Austria) with demineralized water and laboratory washing agents ProCare Lab 10 MA and ProCare Lab 30 C (Miele), rinsed twice with ultrapure water and stored at 180 °C for 16 h. Elution tubes were boiled in ultrapure water for 2 h, subsequently soaked in IPA for 16 h, and dried covered with cleanroom wipes (Dastex, Muggensturm, Germany) in a clean fume hood.

### Experimental setup and sampling

**Fig. 1:**
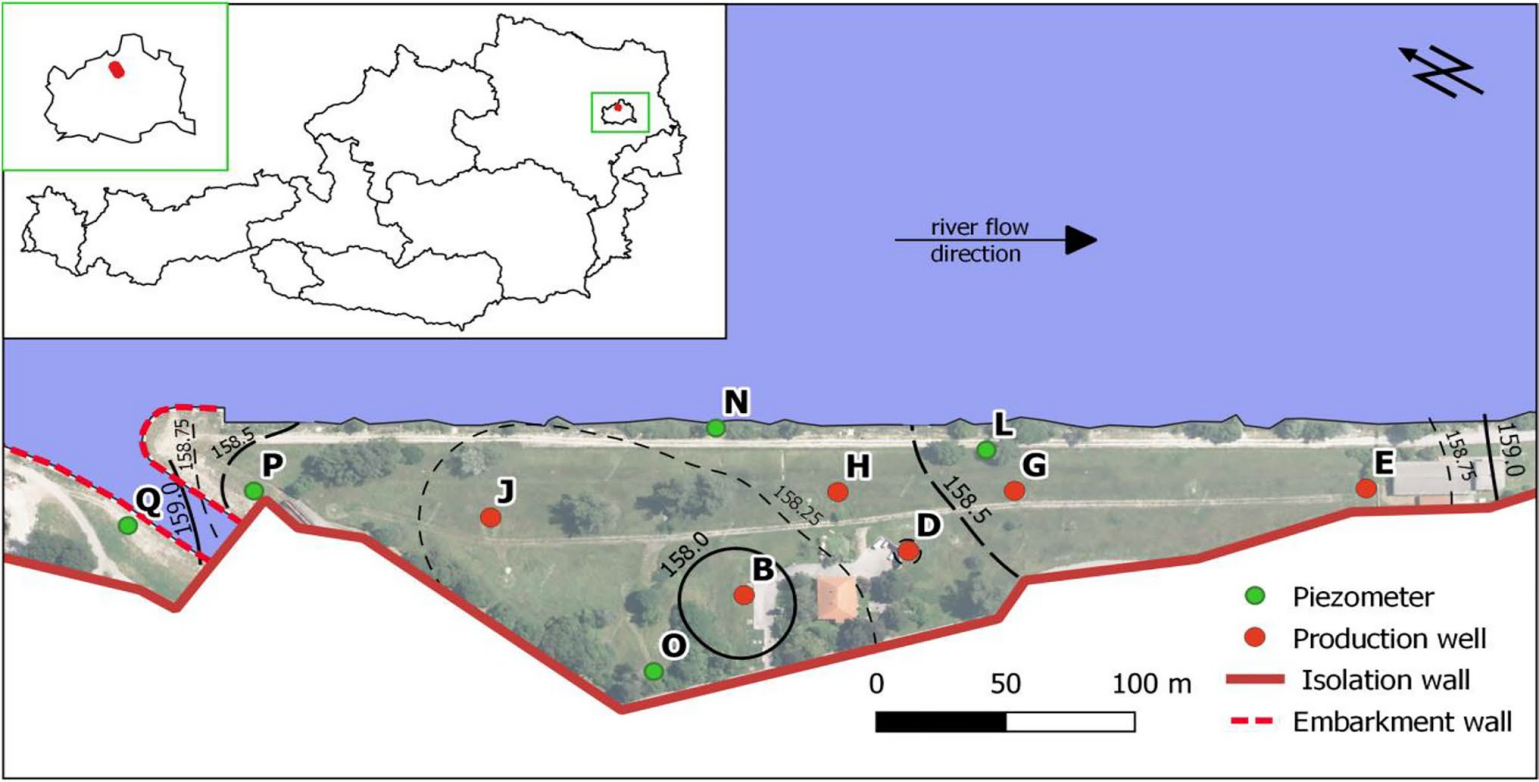
Map of the investigated riverbank filtration site

The sampling location is a 32,000-m^2^ riverbank filtration site at the river Danube where any inflow of native groundwater from southwest aquifer is prevented by an isolation wall (Fig. 1). Sampling location Q is separated from the rest of the well field by a shipping channel. The embarkment walls of the channel and its intake are constructed via bore pile walls, which do not reach the aquiclude. The groundwater flow under the shipping channel and toward the sampling location P from the north and northeast is therefore not prevented completely but significantly impeded. Drilling profiles indicate a very homogeneous aquifer consisting of sandy gravel. Six production wells (sampling locations B, D, E, G, H, J) and five groundwater piezometers (sampling locations L, N, O, P, Q) were chosen for the sampling of groundwater.

Surface water samples from the river Danube were collected using a permanent sampling installation. At sampling location B, the samples were taken from the installed sampling tap fed by the production pumps of the well. Samples at all other sampling locations were pumped from the wells and gauges through a purpose-built stainless-steel pipe using a submersible pump (Grundfos MP1, Brookshire, USA). Before collecting the water samples, the pump was run for 5 min at high flow rate (0.5 L s^−1^) to flush the gauge, pump, and pipe. After flushing, the glass sampling bottle was rinsed. The conductivity, redox potential, and dissolved oxygen were measured on site using TetraCon® (WTW, Weilheim, Germany) 925, SenTix® ORP-T 900 (WTW), and FDO® 925 (WTW) sensors, respectively, which were connected to a multi-parameter portable meter (WTW). Procedural blanks including sampling were prepared by pumping ultrapure water on site with the sampling equipment used for the groundwater gauges and production wells. Before the collection of the procedural blank, approximately 20 L of ultrapure water was used to flush the pump and the pipe.

After sample collection, samples were transported to the lab within 6 h and filtered using a 0.45-µm cellulose filter (PALL-Life Sciences, Vienna, Austria), which were previously conditioned with 90 °C ultra-pure water, within 5 h of arrival. Samples were filtered into 20-mL glass bottles (plastic screw cap with Teflon inlet) and stored at − 20 °C until further analysis.

#### Setup of the spatial study

For the non-targeted spatial study, groundwater samples were taken from 11 groundwater sampling locations in the riverbank filtration site at the river Danube (sampling sites are depicted in Fig. 1). A total of 0.5 L of groundwater from each sampling location was sampled on 2nd May 2017. Abstraction from the well field was 350 L s^−1^ in total during sampling with 150 L s^−1^ from well B and 66.6 L s^−1^ each from wells D, E, and J. The pumping started two weeks prior to the sampling with steady-state flow conditions during sampling. The contour lines in Fig. 1 represent the piezometric surface in the well field during the sampling. Three independently prepared solid-phase extraction replicates from each 0.5 L groundwater sample were analyzed.

#### Setup of the temporal study

For the non-targeted investigation of the temporal study, groundwater samples were taken from the production well at the sampling location B. River Danube water (**C**) and groundwater samples (**B**) were sampled at 25 time points over a period of one year (Table 1). Six of these time points 15C/B, 16C/B, 18C/B, 19C/B, 20 C/B, and 21 C/B were selected with a shorter interval of two to three days maintaining constant pumping regime. Sampling dates were selected following two strategies. First, equidistant sampling over the whole study period with intervals of about 2–3 weeks should give a general overview over the whole hydrological year and seasonal changes. Second, for a period of 11 days in November 2016, the intervals were reduced to 2–3 days to show short-term changes. Due to operation requirements, it was not possible to carry out sampling on some of the planned sampling dates which led to longer intervals in July and August 2016.

**Table 1:** Dates of river (C) and groundwater (B) sampling for the temporal study of the riverbank filtration process

ID	Date	ID	Date	ID	Date	ID	Date	ID	Date
2C/2B	10/10/16	18C/18B	18/11/16	25C/25B	21/12/16	34C/24B	15/03/17	82C/82B	04/07/17
7C/7B	21/10/16	19C/19B	21/11/16	26C/26B	11/01/17	36C/36B	22/03/17	83C/83B	31/08/17
10C/10B	02/11/16	20C/20B	23/11/16	27C/27B	18/01/17	37C/37B	29/03/17	84C/84B	13/09/17
15C/15B	14/11/16	21C/21B	25/11/16	29C/29B	01/02/17	80C/80B	31/05/17	88C/88B	19/09/17
16C/16B	16/11/16	23C/23B	07/12/16	32C/32B	22/02/17	81C/81B	28/06/17	94C/94B	27/09/17

### Sample preparation

Groundwater, surface water, and procedural blank samples were thawed at room temperature and centrifuged in glass centrifuge tubes for 5 min at 2300 rcf. A total of 18.9 mL of each sample was transferred using a Gilson GX-271 ASPEC® (Middleton, USA) liquid handling instrument and spiked with 100 µL of internal standard A (nicotinamide and ααα-trifluoro-*m*-toluic acid, *c* = 8.5 µmol L^-1^) using an air displacement pipette. Subsequently, samples were mixed and SPE was performed using a Gilson GX-271 ASPEC® (Middleton, USA) and 60 mg 3 cc Oasis HLB cartridges (Waters, Vienna, Austria). The cartridges were preconditioned with 3 mL MeOH followed by 3 mL LC–MS grade water. Then, 18 mL of sample/blank containing the internal standards was loaded at a flow rate of 3 mL min^−1^. The cartridges were washed with 250 µL LC–MS grade water and dried under 1 bar nitrogen for 1 min. Analytes were eluted with 3 mL MeOH. The eluates were spiked with 150 µL of internal standard B (norleucine and naringenin, *c* = 4 µmol L^−1^) before drying in a centrifugal vacuum concentrator (GeneVac®, Warminster, USA) and stored at − 80 °C. SPE blank samples were prepared by performing all the steps above using LC–MS grade water. Prior to measurement, all samples (except for QC samples) were reconstituted in 150 µL 0.1% formic acid. QC samples were reconstituted in 300 µL 0.1% formic acid to have sufficient volume for both positive and negative ionization modes. All samples were shaken with IKA® VXR B orbital shaker (Staufen, Germany) first for 3 min at 1500 rpm then for 30 min at 750 rpm.

### HPLC-TOFMS platform

Liquid chromatography was performed using a 1290 Infinity II LC system (Agilent Technologies, Santa Clara, USA) coupled with an ACQUITY UPLC® HSS T3 (1.8 µm, 2.1 × 150 mm) column (Waters) and an ACQUITY UPLC® HSS T3 (1.8 µm, 2.1 × 5 mm) pre-column (Waters). After injection from a fixed 5 µL loop, gradient elution with 0.1% formic acid and MeOH at a constant flow rate of 0.200 mL min^−1^ was performed at 40 °C using the following program: 0% MeOH for 1 min, increased to 70% MeOH in 6 min, and to 100% MeOH in 4.5 min. After 1.5 min of isocratic elution (washing), the MeOH was reduced to 0% (initial mobile phase composition) in 0.1 min, and the column was equilibrated for 5.9 min. The total run time was 19 min.

For mass spectrometric detection, a 6230B LC-TOFMS system (Agilent Technologies) equipped with an Agilent Jet Stream interface was used. Electrospray ionization parameters were set as follows: 180 °C drying gas temperature, 10 L min^−1^ drying gas flow, 35 psig nebulizer pressure, 350 °C sheath gas temperature, 12 L min^−1^ sheath gas flow, 3500 V capillary voltage, and 120 V fragmentor voltage. The TOF detector was operated in the 2 GHz extended dynamic range mode with an acquisition rate of 2.5 spectra per second (accumulation of 5361 TOF transients per spectrum). Spectral data were recorded over a mass range of 90–1700 m/z. During the measurement, a solution containing reference compounds with m/z 121.0509, 922.0098 (positive ionization mode), and m/z 112.9856, 966.0007 (negative ionization mode) (Agilent PN: G1969-85,001) for mass drift correction were introduced using a flow rate of 0.050 mL min^−1^ through a reference sprayer via an Agilent G2226A nanoflow pump (Agilent Technologies).

In the temporal study, the measurements took approximately 27 h per batch. In order to minimize the influence of long-term drift caused by a range of physicochemical effects and instrument contamination, the column effluent was directed to the waste between 0–2.5 min and 13.0–19.0 min while the ESI interface was rinsed with 0.1% formic acid and methanol, respectively.

### Data processing workflow

Following initial checking of the data quality, a non-targeted data evaluation workflow was performed beginning with a batch-recursive molecular feature extraction (rMFE) method in MassHunter Profinder version B10.00 SP1 (Agilent Technologies). For both studies, the integration of all molecular features was controlled visually and peaks were integrated manually when necessary. Additional filtering steps were performed by exporting the peak tables into the csv format for further filtering steps (see the “Compound filtering” section). The data processing workflows were developed according to the requirements of the two primary research questions and the number and types of samples addressed.

#### MassHunter Profinder parameters

For the spatial study, the rMFE method was configured to search for putative compounds eluting between 3.0 and 13.0 min with spectral intensities of ≥ 30 counts via peak picking. In positive ionization mode, protonated molecules and sodium adducts were chosen as putative associated ion species for intrasample alignment, while deprotonated molecules and formate adducts were allowed for the negative ionization mode. For inter-sample alignment of putative compounds, the retention time tolerance was ± 0.30 min and the m/z tolerance was ± (10 ppm + 2.00 mDa). Putative compounds which were detected at least in 2 out of 3 replicates of one sample and scored above 75 (MFE score) were further processed in the recursive feature extraction step. After recursive feature extraction, only compounds which were detected in all measured replicates and scored above 75 (rMFE target score) were accepted, automatically integrated, and passed to the next stage of data processing.

For the temporal study, most of the peak picking settings were retained from the spatial study. The only modification was the required number of detections in post processing filters. In the temporal study, all features, which were detected in a single sample and scored above 75, were processed in the recursive feature extraction step. After recursive feature extraction, only compounds scored above 75 were accepted, automatically integrated, and passed to the next stage of data processing.

#### Compound filtering

Data from the initial Profinder workflow were subjected to several filters in order to reduce the number of artifacts and false positive results.

Erroneous peak picking and alignment results were identified and eliminated using the programing language *R* (R Core Team 2016). Such errors may result from true data artifacts (e.g., detector ringing) or arise from the sensitivity of the peak picking algorithm to noise when dealing with large concentration ranges across multiple samples. In this step, compound groups were firstly built for putative compounds within a mass tolerance range of 10 ppm ± 2.00 mDa and a retention time tolerance of ± 0.1 min. Consequently, all putative pairs of compounds within a group were examined with the following two-step strategy. First, for each of the compound pairs, a linear regression model was applied to compare the intensities across all samples. For this step, missing values were treated as zero values and a *p*-value of the regression was calculated. Second, the compounds were compared according to their simultaneous presence or absence. If both compounds were present or absent in a single sample, they were considered as congruent, if not, they were considered as not congruent. Finally, compounds in each pair were considered to be probable artifacts if the *p*-value of the linear-regression was lower than 0.05 and occurrence was congruent for ≥ 85% of samples. Subsequently, only the compound with the highest median abundance value in a cluster of probable artifacts was retained for further data processing, whereas all other compounds in the cluster were removed from the dataset.

Background correction/elimination was performed using an initial application of a peak area abundance cut-off of 1000 to remove entries of compounds with poor ion counting statistics from any sample. After the abundance cut-off, the results of QC-samples were analyzed separately (see below). The maximum intensity of each compound across the sample batch was assessed and any compound with a maximum abundance < 5000 counts was removed from the dataset. Additionally, for all compounds detected in instrumental, SPE, or procedural blanks, an LOQ (limit of quantification) was calculated based for each background sample type (solvent blank, SPE blank, procedural blank) utilizing the average (AV) and the standard deviation (SD) (Eq. (1)) (Shrivastava and Gupta 2011).

1 $$LOQ=10\times {SD}_{blank}+{AV}_{blank}$$

Thus, entries for compounds in all individual samples except the QC-samples with abundances lower than the highest of the three LOQs were removed (i.e., some compounds were found to have higher abundances in the instrumental blank than in the SPE blank and procedural blank).

In addition, in the spatial study, intensities were set to 0 if they were detected in only one of the three sample replicates of a sampling location. Finally, all compounds and their abundances within samples that were retained after these filtering steps were considered as “substantiated,” i.e., as relevant for further interpretation.

For assessment of signal intensity drift, data acquired from QC samples were analyzed across the sample batch. For each compound detected in all replicates with a maximum abundance > 5000 counts, signal intensity drift was calculated as follows (Eq. (2)).

2 $$Signal\;intensity\;drift\;\left[\%\right]=\left(1-\frac{Minimum\;intensity}{Maximum\;intensity}\right)\;\ast\;100$$

## Results and discussion

### Quality control assessments

Analytical non-targeted studies are lacking certified reference materials and are therefore facing a significant challenge regarding method validation. Sources of uncertainty may be variable for the 100 s or even 1000 s of putative compounds evaluated across multiple samples and, often, multiple batches (Sangster et al. 2006; Dunn et al. 2012; Dudzik et al. 2017; Broadhurst et al. 2018). Pooled QC samples are well established, enabling the continuous assessment of analytical variations (e.g., signal intensity drift) of LC-HRMS sequences and possible application of mathematical or statistical corrections within a single batch or across multiple batches. In general, compounds which are introduced unintentionally (“blanks”) by sampling, sample processing, and the analytical workflow need to be carefully assessed and considered during data evaluation and interpretation. Specifically, the use of SPE as a part of the sample preparation in non-targeted workflows demands for careful assessment of repeatability precision (e.g., via internal standardization) and a post hoc elimination of compounds introduced from the SPE material itself. Both aspects were recognized as the largest source of analytical uncertainty for the presented workflow, as erroneous conclusions may be drawn by under-/over-estimation of the uncertainty of the entire procedure and by inclusion of false positive results generated by the “bleeding” of unwanted compounds from the SPE cartridge. To this end, instrument blanks, SPE blanks, procedural blanks, and QC samples were included as part of this study to assess the suitability of the developed methods for addressing the goals of the study.

#### Precision under repeatability conditions of measurement of entire analytical workflow

Two different internal standard mixtures, each containing two internal standard substances each (see the “Material and methods” section), were used for assessment of sample preparation and LC-TOFMS measurements. Results for internal standard A (nicotinamide and ααα-trifluoro-*m*-toluic acid), which was added prior to SPE, were used to determine the precision under repeatability conditions encompassing SPE and all subsequent steps of the analytical procedure. Internal standard B (norleucine and naringenin) added after SPE step was used for the independent assessment of the post-SPE steps of the workflow. Based on their ionization characteristics in electrospray, nicotinamide (added before SPE) and norleucine were used for the positive ionization mode (added after SPE), while ααα-trifluoro-*m*-toluic acid (added before SPE) and naringenin (added after SPE) were used for the negative mode.

Naringenin was found not to be suitable as internal standard as it showed high relative standard deviation in all measurements in negative ionization mode (see Table 2), which is most probably due to the poor solubility in 0.1% FA which was used for reconstitution of the evaporated SPE fraction. The relative standard deviation of the three other internal standards (Table 2) obtained under repeatability conditions of measurement indicate good precision of both the SPE procedure and the LC-TOFMS measurement.

**Table 2:** Relative standard deviation of internal standards signals obtained from all samples (temporal and spatial study)

Study	%RSD in positive ionization mode			%RSD in negative ionization mode		
	Number of injections	Nicotinamide	Norleucine	Number of injections	ααα-Trifluoro-m-toluic acid	Naringenin
Spatial	33	10.0%	13.4%	33	10.7%	58.6%
Temporal	50	10.5%	8.3%	50	12.8%	55.9%

#### Pooled QC assessment

For both studies, pooled QC samples were used for monitoring of effects occurring during long measurement sequences. Pooled QC samples from one single SPE were measured 6 times (technical replicates), at the beginning, after every 15th sample, and at the end of the sequence. Measurement drift was assessed using the median drift per hour determined for compounds that were substantiated for the QC samples (Table 3). The assessment revealed a compound-dependent signal intensity drift. The median drift of all compounds detected in the four sequences was lower than 2% signal intensity per hour. In the worst case (spatial study, positive ionization mode), this resulted in a median signal loss of 47% during a 24-h sequence. Directing the column effluent to waste at the beginning and at the end of the LC run had a beneficial effect on the intensity drift obtained during the analysis of the samples from the temporal study (see Table 3).

**Table 3:** Median signal intensity drift for all four measurement sequences calculated from the substantiated compounds detected in QC samples (*n* = 6)

Sequence name	Ionization mode	Sequence duration	Median drift per hour
Spatial study	Positive	24 h	1.96%
Spatial study	Negative	24 h	1.38%
Temporal study	Positive	26.3 h	1.30%
Temporal study	Negative	26.3 h	0.73%

#### Effect of compound filtering steps

**Fig. 2:**
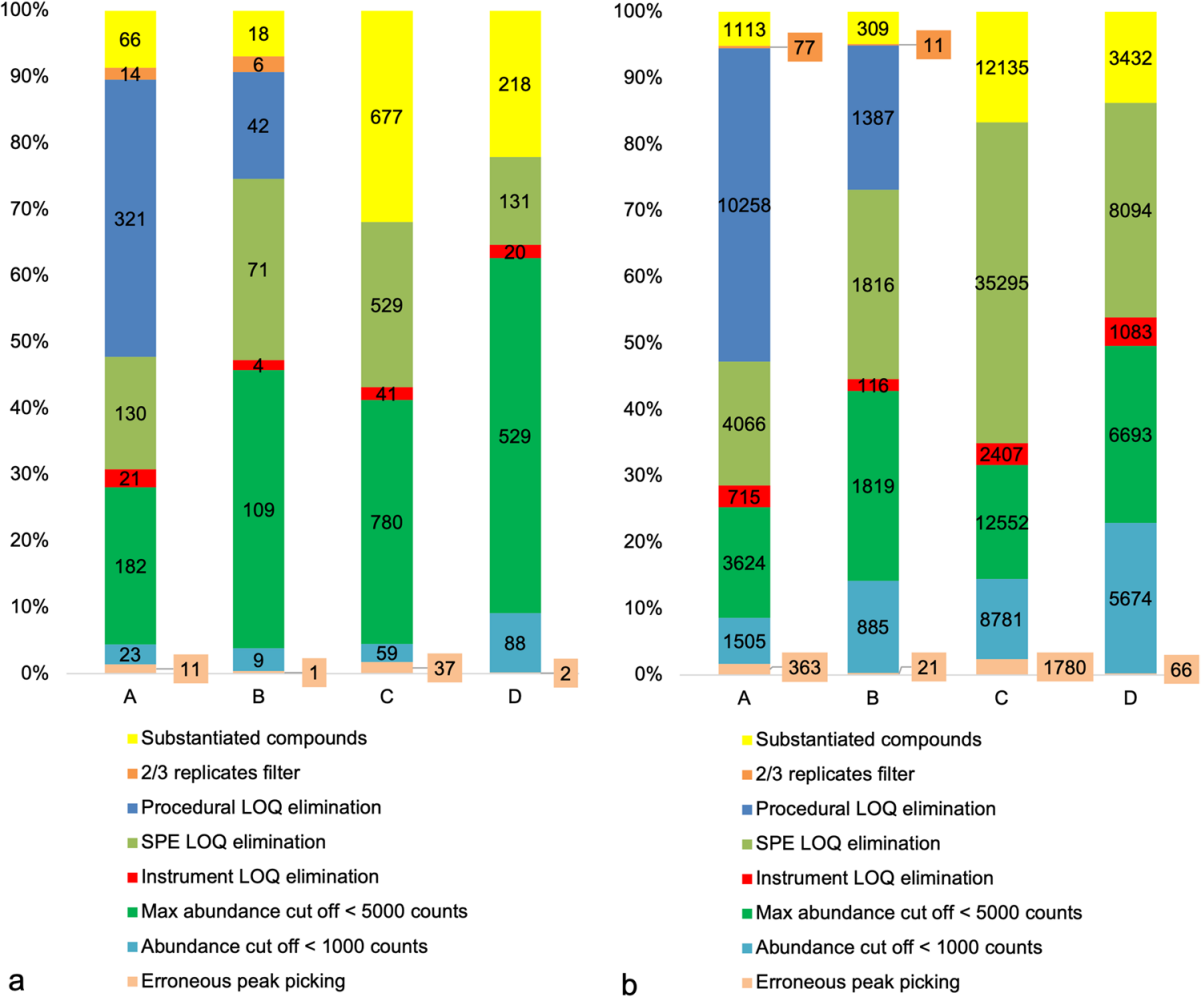
Effects of compound filtering steps in NTA of water samples. **a** Percentage and number of compounds classified according to the applied filtering step; **b** number of single detections from every sample classified according to the applied filtering step; (A) spatial study, positive ionization mode; (B) spatial study, negative ionization mode; (C) temporal study, positive ionization mode; (D) temporal study negative ionization mode. It is noteworthy that the low compound number in spatial analysis **a** and **b** is due to the sample collection from 11 sampling locations on a single day, while in temporal analysis, 25 samples were collected in one year

The application of the filtering and elimination steps as described in the “Compound filtering” section reduced the number of substantiated compounds by between 68 and 93% considering both studies and ionization modes (Fig. 2A). In negative ionization mode, application of the 1000 (compound-level) and 5000 (maximum abundance across samples) cut-off steps had a proportionally higher influence than in positive ionization mode, which is due to the lower sensitivity in negative mode. It is noteworthy that the blank compounds introduced by sampling (procedural blank), sample preparation (SPE blank), and LC–MS (instrument blank) are overall representing a significant percentage of the data eliminated by different filtration steps (15–61%). A large number of single occurrences from all samples via blank elimination steps were removed by this procedure (Fig. 2B). The very high number of single occurrences eliminated by blank elimination highlights the importance of not only preparing procedural blank samples for every step of the non-targeted workflow, but also to report the blank elimination technique employed and to elaborate its influence on the results. As a worst case, without application of such steps, compounds introduced by, e.g., SPE might be erroneously included and identified for environmental monitoring purposes.

### Spatial study of riverbank filtration site

In previous studies, it has been shown that the removal of organic pollutants by riverbank filtration processes depends strongly on the prevailing hydro-chemical conditions and that increasing distance from the source water can improve the removal of organic pollutants (Kruć et al. 2019). Thus, the aim of this spatial study was to compare the molecular composition of riverbank filtrate from different sampling locations across the riverbank filtration site to better understand the homogeneity of the site and to fully test the ruggedness of the developed non-targeted method for a sample set representing all potential sampling locations.

For this study, samples were taken from 11 sampling locations at the riverbank filtration site at the river Danube (Fig. 1). Three independently prepared samples from each sampling location were processed applying the NTA workflow (described above) to improve data quality by reducing false positive hits. Based on the results obtained for the instrumental, SPE, and procedural blanks, the strict filtering criteria applied yielded a low number of substantiated compounds for this assessment. Finally, 66 compounds from positive ionization mode measurements and 18 compounds from negative ionization mode measurements were employed to assess relative differences between the sampling locations and the homogeneity of the well field.

**Fig. 3:**
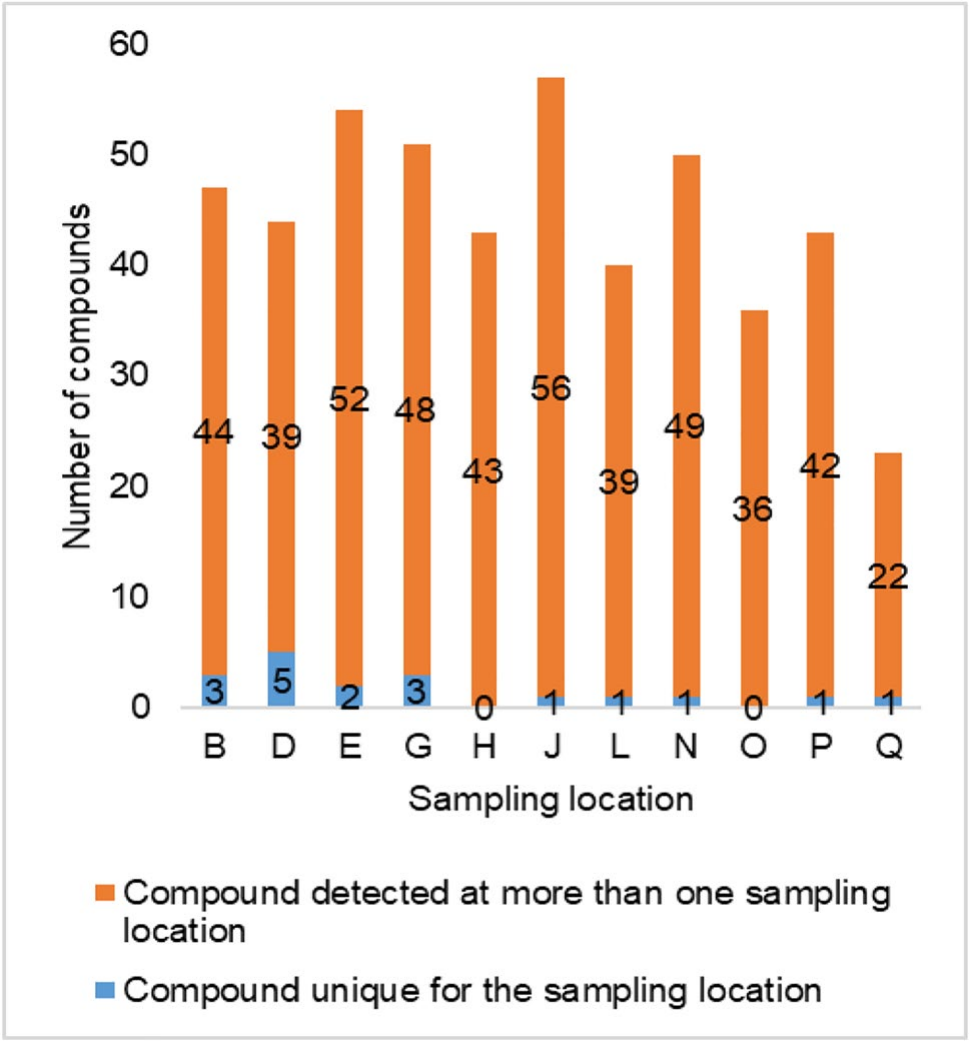
Number of substantiated compounds detected at different sampling locations in spatial study of the riverbank filtration site

**Fig. 4:**
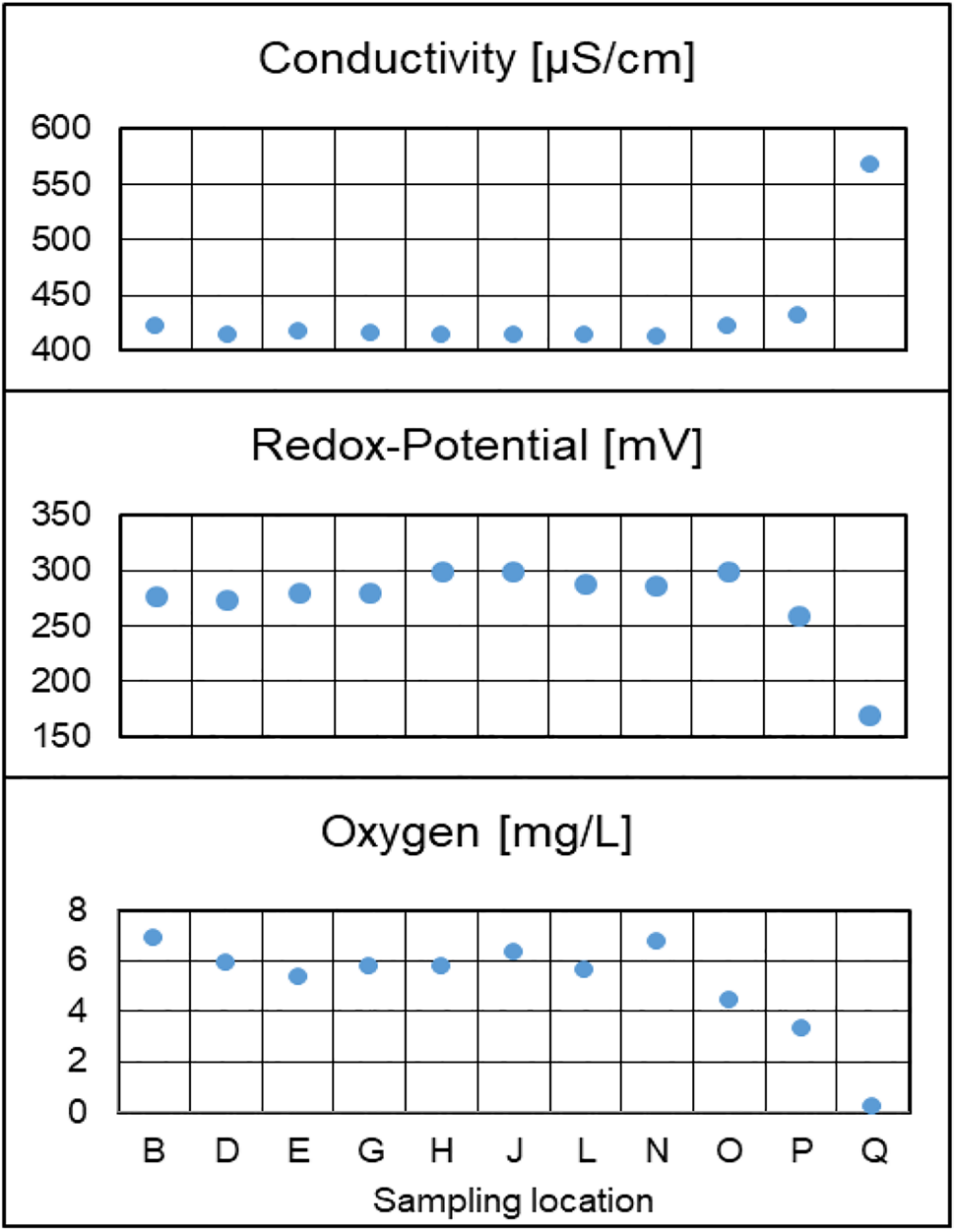
Physicochemical parameters determined in-situ in the investigated groundwater samples. The precision of the conductivity, oxygen, and redox-potential measurement are ± 0.5% RSD, ± 0.5% RSD, and ± 0.2 (mV), respectively

In the spatial analysis of the well field, the majority of substantiated compounds were detected at more than one sampling location (Fig. 3). Only a very low number of unique compounds were detected. The lowest number of compounds was detected at sampling location Q, which is situated between the isolation walls and semipermeable embarkment walls (Fig. 1). Due to the position of sampling locations Q and P, the residence time of the water is increased compared to the other sampling locations. This significantly affected the physicochemical parameters at both sampling locations (Fig. 4). However, due to the higher water flow restrictions, anoxic conditions are prevailing at sampling location Q. This influences the physicochemical and biological processes occurring along the riverbank filtration compartment and explains the observed difference in the number of detected compounds.

Multivariate hierarchical clustering analysis with Euclidean distance metric and average linkage rule was applied for all substantiated compounds across the sampling locations. Figure S1 shows the resulting dendrogram and heatmap. Similar to the results of the physicochemical parameters, the sampling location Q clustered away from the rest of the sampling locations. Other clusters involved sampling locations without any clear correlation with the distance of the sampling locations to the water source. Except at sampling location Q, the riverbank filtration efficiency in the studied well field is relatively homogeneous, which may be explained by the similar distance between the sampling locations and the surface water.

It is noteworthy that the spatial analysis has been performed on samples originating from one single sampling date (2nd May 2017). Evidently, several parameters (e.g., surface water temperature, river water level, pollution load of the river) which affect riverbank filtration efficiency can change depending on seasonal changes or extreme weather events. To evaluate the influence of these parameters on the riverbank filtration site, additional spatial studies need to be conducted over longer period at different time points.

### Temporal study of surface water and groundwater at the riverbank filtration site

A temporal study was undertaken to directly gauge how NTA can be used to characterize surface water and riverbank-filtrate or groundwater for monitoring riverbank filtration efficiency and for assessment of compound residence time in the riverbank filtration compartment. This study required the assessment of the relative quantitative variation of substantiated compounds between samples obtained from surface water (Danube River) and the corresponding riverbank filtrate (groundwater at production well B) at 25 time points over a period of one year. In positive ionization mode, a total of 677 compounds were substantiated with 481 compounds detected in both water compartments, 138 compounds were detected exclusively in surface water, and 58 compounds exclusively in groundwater. In negative ionization mode, a total of 218 compounds were substantiated with 153 compounds detected in both water compartments, 50 exclusively in surface water, and 15 exclusively in groundwater.

The developed non-targeted LC-TOFMS-based workflow including SPE for sample clean up and analyte enrichment is fit-for-purpose to characterize and monitor natural waters in the context with riverbank filtration. It is evident that increasing the sample intake of 18.9 mL to higher volumes will lead to a higher number of detected compounds. In general, the workflow has the advantage that all stored data can be analyzed retrospectively if new priority contaminants emerge in future.

#### Comparison of temporal changes in surface water and groundwater at the well field

**Fig. 5:**
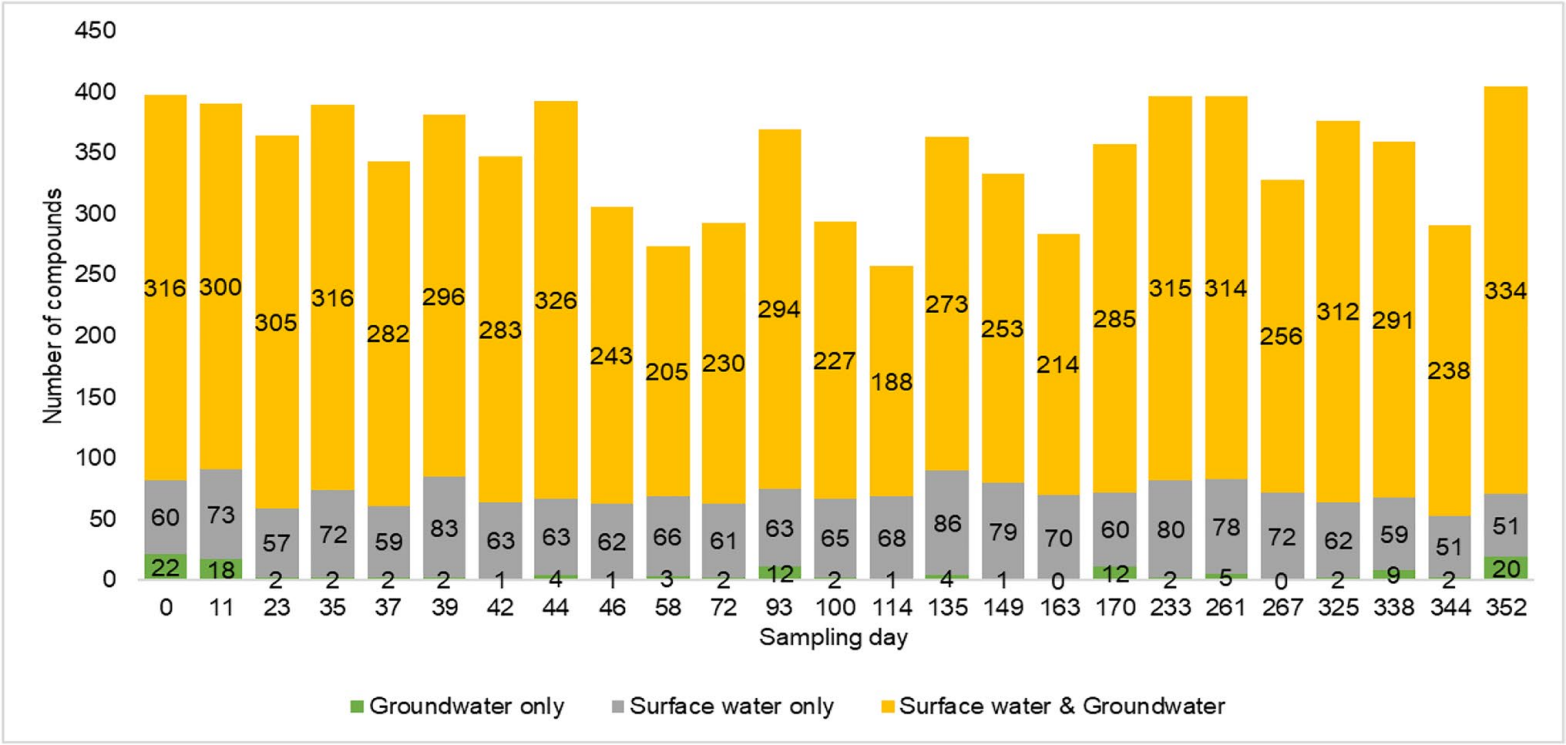
Substantiated compounds detected in the temporal study of the riverbank filtration site (positive ionization mode)

For monitoring purposes, the number of substantiated compounds per sampling day and their intensities were used to understand fluctuations of compounds of natural and anthropogenic origin in the water phase. Figure 5 depicts the number of compounds detected in positive ionization mode at the 25 sampling days in both surface water and groundwater. The sampling days are not equidistant (sampling dates see Table 1). It was observed that a high variation of the total number of detected compounds can occur even over short intervals (e.g., sampling days 44 and 46). Furthermore, the relatively stable fraction of compounds detected exclusively in surface water (13–26%) indicates that the riverbank filtration process was reproducible over the sampling period.

**Fig. 6:**
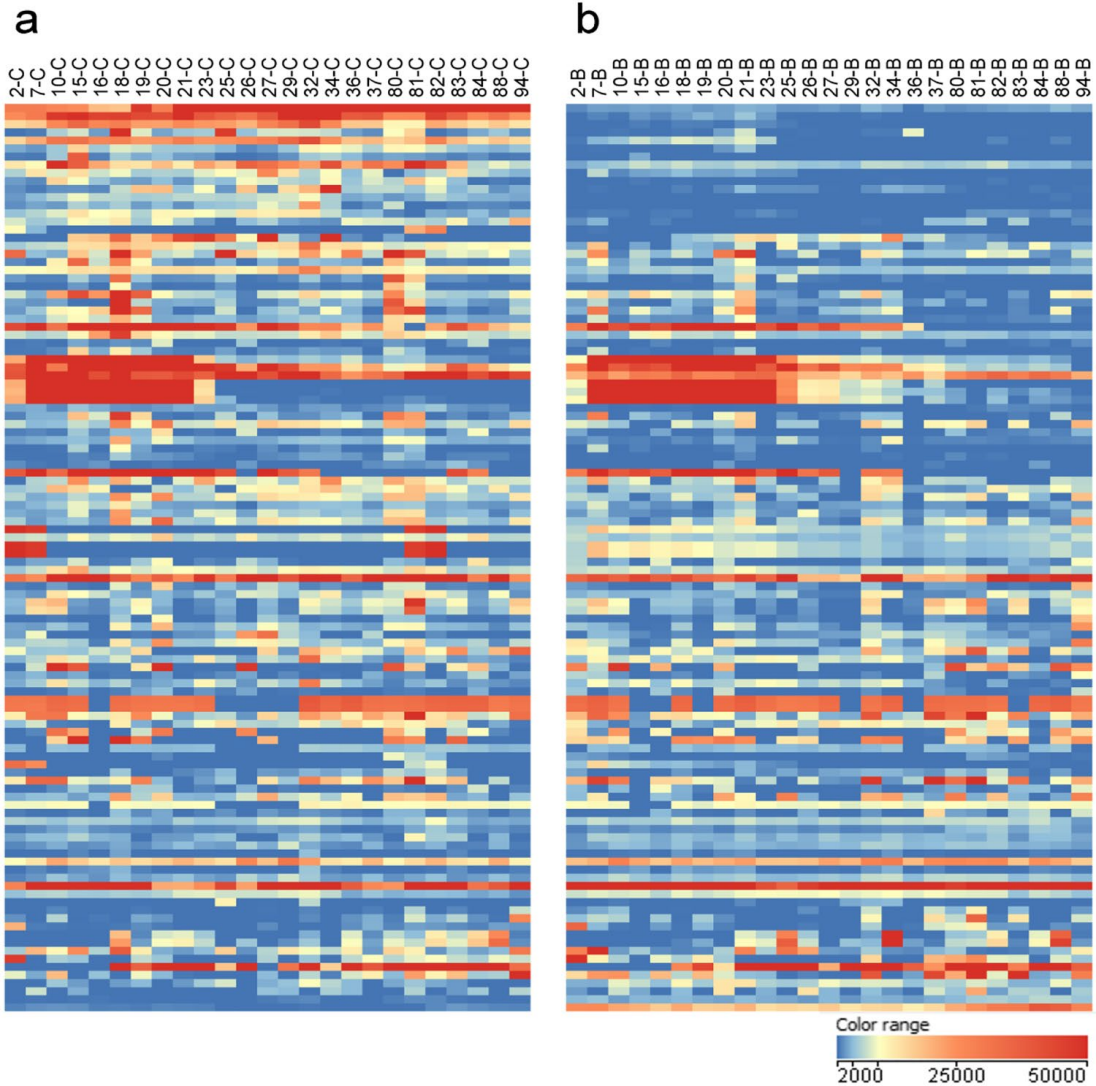
Heat map of 112 substantiated compounds remaining in the dataset after the prioritization step described above (detection in groundwater in at least 60% of the samples). Each column represents a sampling day. Surface water samples obtained from the river Danube (**a**) were compared to the corresponding groundwater samples obtained from the investigated riverbank filtration site (**b**). The color range represents the abundance of the substantiated compounds (blue = low abundant, red = high abundant)

In the temporal study, 481 substantiated compounds present in both water sources were further filtered down to 112 compounds, which were detected in groundwater in at least 60% of the samples. Applying this data reduction step, substantiated compounds which were most frequently penetrating the riverbank filtration compartment should be prioritized for identity confirmation (Fig. 6).

#### Assessment of riverbank filtration efficiency

**Fig. 7:**
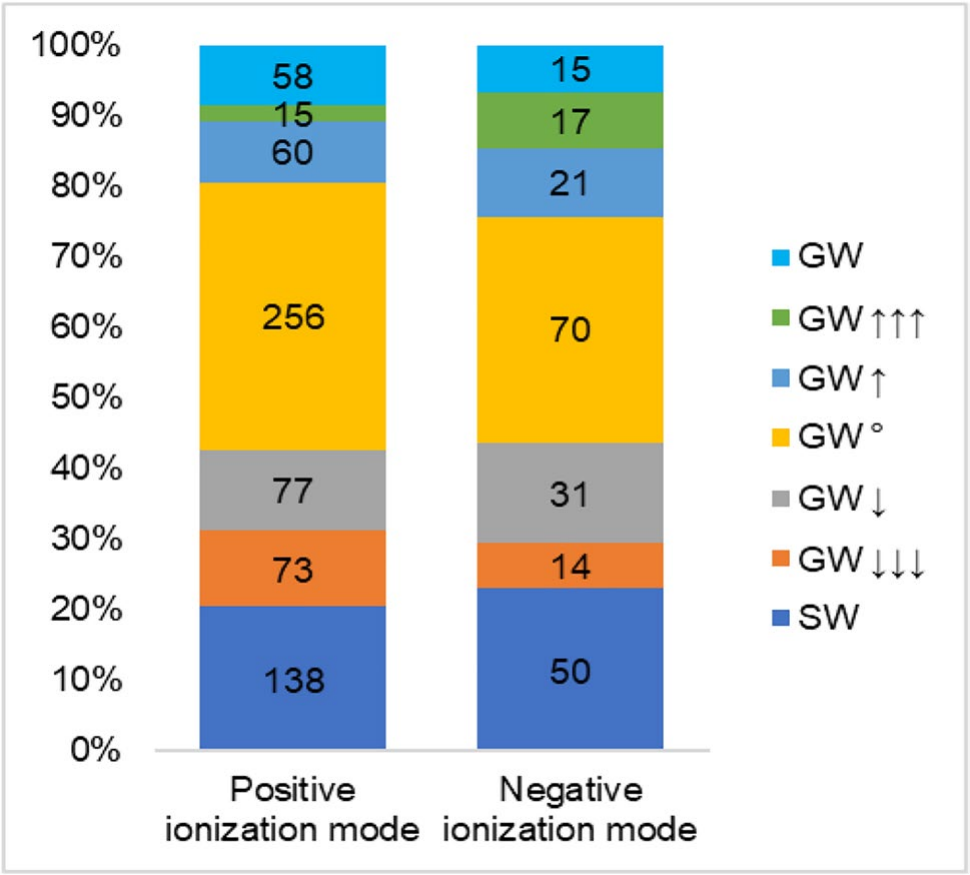
100% stacked column chart of the detected compounds categorized according to relative fold changes between surface water (SW) and groundwater (GW). For interpretation of the legend, see the “Assessment of riverbank filtration efficiency” section

In the view of the impossibility of acquiring riverbank filtration samples which are exactly corresponding to surface water samples in the time domain, the filtration efficiency of the investigated site (well B) was evaluated by comparing data for substantiated compounds in surface water and groundwater over the entire temporal study using the fold change (FC) of the arithmetic mean of the intensities. Depending on their FC introduced by the river filtration process, the substantiated compounds were categorized into seven groups (see Fig. 7). Compounds with an absolute FC < 2 were categorized as “constant” (GW°). Compounds with an absolute FC between 2 and 5 were categorized as “increase/decrease” (GW↑/GW↓), whereas molecular features with an absolute FC > 5 were categorized as “high increase/high decrease” (GW↑↑↑/GW↓↓↓). Compounds found only in surface water and only in groundwater were classified into separate categories (SW and GW, respectively).

After applying this categorization, results of the positive and negative ionization mode were in good agreement with a similar distribution of compounds across all categories (Fig. 7). The evaluation of positive ionization mode data revealed that 138 substantiated compounds were detected exclusively in surface water, indicating that the passage of these compounds had been retarded by the riverbank. Additionally, the concentration of 150 compounds were significantly decreased by riverbank filtration. A total of 58 compounds were detected in groundwater only, and the concentration of 75 compounds was significantly higher in groundwater. The compounds within the latter two categories may be transformation products or soil-related substances emerging from the riverbank. A total of 256 compounds were found to not be significantly affected by the riverbank filtration compartment to the production well. Considering compounds from all the categories other than “constant,” some interpretations can be tentatively made according to the polarity estimations derived from reversed phase chromatography. Below a retention time of 7.5 min, the number of compounds detected with a higher intensity in groundwater was significantly lower than the number of compounds detected with a higher intensity in surface water (Fig. S2). The retention time distribution of compounds indicates on the one hand that the well field provides physical, chemical, and microbiological conditions for removal of compounds over a broad polarity range and on the other hand confirms the abovementioned occurrence of soil-related compounds with lower polarity.

#### Assessment of compound residence in the riverbank filtration compartment

The determination of residence times of compounds passing the riverbank under different hydrological conditions can support the estimation of suitable response times for setting drinking water protection measures. Samples from a sampling campaign with shorter intervals were selected to evaluate the potential of NTA in this context. Sampling was performed on 6 sampling days (see the “Setup of the temporal study” section) with an interval of two to three days maintaining a constant pumping regime. Substantiated compounds which appeared in surface water on 18/11/2016 with at least a fivefold higher abundance than earlier surface water samples were selected as possible indicators. From this group, those appearing in groundwater on 18/11/2016 with a relative abundance of < 20% in surface water on same day were retained. Finally, any compounds with an abundance of < 5000 in groundwater were excluded resulting in a final list of 19 substantiated compounds in positive ionization mode and 7 substantiated compounds in negative ionization mode fulfilling these criteria within the selected time segment. In Fig. S3, data for 6 of these compounds in surface and groundwater are presented. Compounds with high abundances in surface water samples on 18/11/2016 were detected in groundwater samples with an increased intensity on 23/11/2016 or 25/11/2016 indicating that a period of 5 to 7 days was needed for compounds to reach the sampling location under the predominant operating state during the study. The low resolution in sampling frequency and the short sampling interval do not allow for a more accurate interpretation of the dynamics of compound residence, but the results show that the method has the potential for accurate evaluation of the residence time at a higher sampling frequency and over longer sampling periods.

## Conclusions

The spatial study of the 32,000 m^2^ riverbank filtration site resulted in a homogenous chemical composition in the well field with the exception of a sampling location where the physical parameters of the water also differed significantly, due to the long residence time of the water. Moreover, the temporal analysis revealed that the riverbank filtration significantly reduces the intensity of organic compounds and that the residence time of selected compounds was 5–7 days.

It is noteworthy that one single analytical technique, as presented in this work, is not sufficient to allow the full understanding of the riverbank filtration processes. Compound coverage by NTA is generally limited by the employed SPE material, separation, and ionization technique. For a more comprehensive analysis, samples need to be analyzed with a variety of orthogonal analytical techniques (e.g., HILIC-MS or GC-EI-MS) to cover more compounds over a wider polarity range and with different ionization properties. Since the filtration efficiency of riverbank filtration is compound-dependent, different analytical techniques with different compound coverage and detection limits will provide complementary degrees of information.

For NTA of natural water, there is currently no standardized method or procedure available for the filtering and elimination of blank compounds. However, this elimination procedure is identified in this study as a major source of uncertainty in data interpretation via false positive/false negative results. The evaluation, selection, and documentation of this procedure, which is an essential part of the workflow, are critical to assure the reproducibility of studies performed in this field.

Compared to targeted approaches, NTA has the potential of both retrospective data analysis and extensive compound coverage supporting the discovery of unknown degradation and transformation compounds. Cost for non-targeted high-resolution instrumentation is approximately three times more expensive than targeted quadrupole based instrumentation. Moreover, NTA demands for significantly higher computing power and data storage. In addition, complex data evaluation, visualization, and interpretation processes are more time consuming and highly skilled personnel is needed.

## Supplementary Information

Below is the link to the electronic supplementary material.

Supplementary file1 (DOCX 1055 KB)

## Data Availability

The datasets used and/or analyzed during the current study are available from the corresponding author on reasonable request.
