# Supplementary material for: Non-targeted analysis with high-resolution mass spectrometry for investigation of riverbank filtration processes
Source: Environ Sci Pollut Res Int. 2022 Apr 26;29(43):64568–81. doi: 10.1007/s11356-022-20301-2 (PMC9481508; doi:10.1007/s11356-022-20301-2)
Supplement: Supplementary file 1 — Supplementary file1 (DOCX 1055 KB) [file 11356_2022_20301_MOESM1_ESM.docx]

# Non-targeted analysis with high resolution mass spectrometry for investigation of riverbank filtration processes

Kaan Georg Kutlucinar^1,2^, Sebastian Handl^2^, Roza Allabashi^2^, Tim Causon^1^, Christina Troyer^1^, Ernest Mayr^2^, Reinhard Perfler^2^, Stephan Hann^1^*

^1^ Department of Chemistry, Institute of Analytical Chemistry, University of Natural Resources and Life Sciences, Vienna, Muthgasse 18, 1190 Vienna, Austria

^2^ Department of Water, Atmosphere and Environment, Institute of Sanitary Engineering and Water Pollution Control, University of Natural Resources and Life Sciences, Vienna, Muthgasse 18, 1190 Vienna, Austria

**corresponding author: stephan.hann@boku.ac.at, Telephone:+43 1 47654-77000*


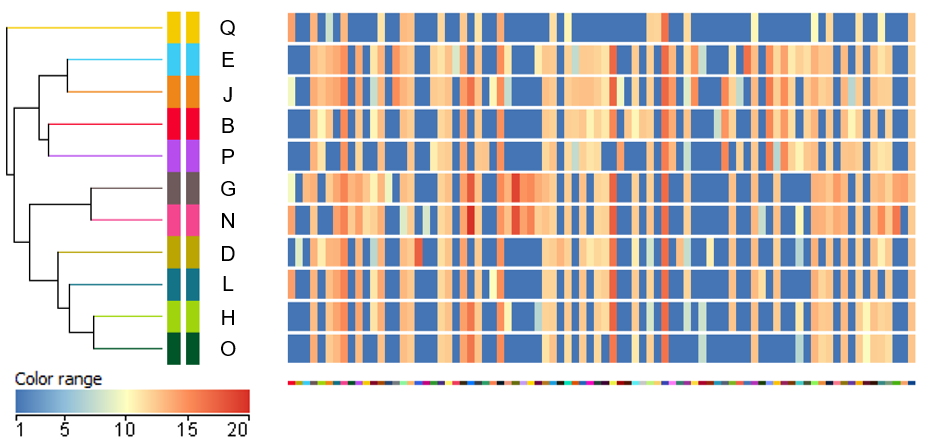


Figure S1. Multivariate clustering analysis of substantiated compounds detected in the groundwater of the sampling locations at the investigated well field using Euclidean distance metric and average linkage rule. The color range uses the log2 abundance values.


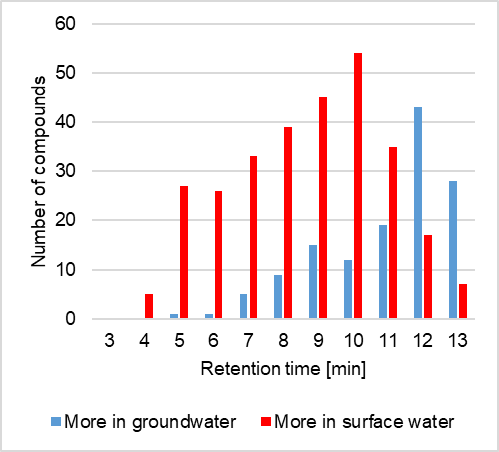


**Figure S2.** Histogram of chromatographic retention times of compounds with significantly higher intensities in groundwater (blue columns) or surface water (red columns)

Figure S3. Abundances of selected compounds detected in samples taken with a higher frequency (i.e., every 2-3 days) for the estimation of an approximate passage duration from surface water to groundwater sampling location.
